# Supplementary material for: Prevalence, acceptability, and cost of routine screening for pulmonary tuberculosis among pregnant women in Cotonou, Benin
Source: PLoS One. 2022 Feb 22;17(2):e0264206. doi: 10.1371/journal.pone.0264206 (PMC8863221; doi:10.1371/journal.pone.0264206)
Supplement: Attachment — Submitted filename: Review.docx [file pone.0264206.s002.docx]

**Manuscript Number PONE-D-21-23077**

**Prevalence, acceptability and cost of routine screening for pulmonary tuberculosis**

**among pregnant women in Cotonou, Benin**

Thank you for asking me to review the manuscript ‘Prevalence, acceptability and cost of routine screening for pulmonary tuberculosis among pregnant women in Cotonou, Benin.’ In this mixed-methods, cross-sectional study with a cost assessment, the authors evaluate the yield, cost, feasibility, and acceptability of routine tuberculosis (TB) screening of pregnant women in Cotonou, Benin. The manuscript is well-written and on the whole reads easily. However, I have some comments below which do need to be addressed before this manuscript can be published.

**Main Comments**

**1. Prevalence rate**

1.1. The authors’ report a TB prevalence rate of 49 per 100,000 among pregnant women enrolled in their study. However, in this cross-sectional study ~ 4000 pregnant women were screened only once during their pregnancy for TB, and not throughout their pregnancy. In addition, only those who had a cough longer than two weeks were asked to provide sputum for analysis. So, in fact, are the authors not are reporting a prevalence rate of 49 per 100,000 for one visit amongst those who had a cough and for whom a sputum sample was sent? For a TB prevalence rate among pregnant women, all ~ 4000 pregnant women would need to be screened and tested at each and every ANC visit throughout their pregnancy. The authors do acknowledge in the limitations that participants were only screened once during their pregnancy, but the authors need to clarify that they are reporting a prevalence rate of 49 per 100,000 for one visit amongst those who had a cough and for whom a sputum sample was sent.

1.2. Given the limitations in using only two cases of TB to determine a prevalence rate, a more robust discussion of the sensitivity and specificity of a cough of longer than two weeks and GeneXpert would be helpful. Given the increasing awareness of asymptomatic TB, this must be addressed in the discussion.

**2. The death of the infant in one of the women diagnosed with TB.**

In the all-important first paragraph of the discussion the authors write ‘the benefit of early detection and prompt treatment is apparent, given the adverse fetal outcome among one woman diagnosed with TB.’ The implication is that the infant’s death was TB-related. However, no evidence is given confirming this. Was the infant diagnosed with congenital TB? Were congenital or other morbidities excluded?

**3. Reporting of cost analysis**

Once published, this article will most probably be read by people who work in TB, many of whom may not be familiar with the technical terms used to describe the cost analysis. Simpler more accessible terminology should be used for the following:

‘purchasing power parity’

‘material costs’

‘accessioning’

‘annuitized’ – the term annualised is more intuitive.

‘prorated’

‘realized costs’

**4. The reporting of the qualitative results** (Page 10)

‘All pregnant women interviewed supported TB screening during routine antenatal care visits and

mentioned its usefulness in preventing pregnancy complications. All midwives also found routine

TB screening during antenatal care visits to be very useful…’

This very generalised reporting of the qualitative results is inadequate and more detail is needed to substantiate the findings reported.

- What do you mean pregnant women supported TB screening? What was said in the interviews that led you to this conclusion?
- What pregnancy complications did pregnant women say could be avoided by screening?
- What did the midwives say that led the authors to conclude that screening during ANC visits was useful?

**Minor Comments**

**Page 3:**

The sentence ‘Additional factors such as human immunodeficiency virus (HIV) and diagnosis of advanced disease at late term in pregnancy are also associated with poor prognosis in pregnant women with TB.’

This sentence is unclear, are you referring to advanced TB or advanced other diseases?

**Page 4:**

‘…..located within a radius of 15km from the NTP and included three public…..’

Do you mean the NTP offices?

‘Consecutive women…’ Do you mean all women?

‘Included women were between the ages of 14 and 45 years and had a pregnancy test, an ltrasound, or a gynecological examination certifying pregnancy…..’

Were these your inclusion and exclusion criteria? If so clarify this.

**Page 5:**

‘Taking into consideration midwife workload, we asked them to at least focus on the most important sign which is the presence of a cough for more than 2 weeks.’

After reading this sentence I was surprised to read in the results section that you did ask and record other symptoms. Maybe you could clarify in the text exactly what was done, something like: 'Having recorded all TB symptoms, the questionnaire focussed on coughs of more than 2 weeks.’

**Page 6:**

‘All participants were randomly selected.’

How were participants randomly selected?

‘The number of participants was determined *a priori*.’

On what basis was this *a priori* decision made.

‘These interviews were conducted onsite but separate from antenatal care rooms.’

What is of importance and interest is whether the interviews were conducted in a confidential place where the participant could not be overheard.

**Page 9**

‘Weight loss/stagnation’

By stagnation do you mean a failure to gain weight?

Page 12

‘….33% were premature…’

This is unclear, you need to clarify that this was 33% of the infants.
