# Supplementary material for: Prevalence, acceptability, and cost of routine screening for pulmonary tuberculosis among pregnant women in Cotonou, Benin
Source: PLoS One. 2022 Feb 22;17(2):e0264206. doi: 10.1371/journal.pone.0264206 (PMC8863221; doi:10.1371/journal.pone.0264206)
Supplement: Attachment — Submitted filename: Response to Reviewer Comments_R2.docx [file pone.0264206.s004.docx]

Dear Dr. Emma Kalk,

Thank you for taking the time to review our revised manuscript and providing further comments to improve it. We have responded to each of your comments in order using **bolded, red text**. We believe we have submitted an improved manuscript and hope you agree.

Many thanks,

Menonli Adjobimey and Jonathon Campbell, on behalf of all authors

**Comments**

1. Both reviewers 1 and 2 noted that screening was conducted at a single time-point during pregnancy. This has been included as a limitation. However, your response to Reviewer 2 point 7 is incomplete: Methods – line 186 on the in the tracked document: The study design is cross-sectional with screening at a single time-point per participant. These data shouldn’t be used to calculate the TB incidence over 9 months for your sample and you are unable to make the comparison with TB incidence over 9 months from the national data (risk difference). You could drop this analysis and amend the relevant lines in the discussion and conclusion.

**We have revised the manuscript to remove this comparison. We have also added 95% confidence intervals to our prevalence estimate, to give a sense of uncertainty. We have specifically removed any mention of the analysis from our methods and results. In the discussion, we have revised wording of paragraph 3.**

2. Reporting qualitative results: please indicate whether the quoted responses are from a pregnant woman or midwife.

**We have updated the quotations with indications of who gave the response. We specifically now state “Original Participant Response” and “Original Midwife Response”.**
