# Supplementary material for: Prevalence, acceptability, and cost of routine screening for pulmonary tuberculosis among pregnant women in Cotonou, Benin
Source: PLoS One. 2022 Feb 22;17(2):e0264206. doi: 10.1371/journal.pone.0264206 (PMC8863221; doi:10.1371/journal.pone.0264206)
Supplement: Attachment — Submitted filename: Response to Reviewer Comments.docx [file pone.0264206.s003.docx]

Dear Dr. Emma Kalk,

Thanks to you and the reviewers for taking the time to review our manuscript and providing helpful comments to improve it. We have responded to each editor and reviewer comment in order using **bolded, red text**, and where appropriate have referenced where changes in our manuscript have been made in the **clean version** of the manuscript. We believe we have submitted an improved manuscript and hope you agree.

Many thanks,

Menonli Adjobimey and Jonathon Campbell, on behalf of all authors

**Editor Comments**

1. Please ensure that your manuscript meets PLOS ONE's style requirements, including those for file naming. The PLOS ONE style templates can be found at
   https://journals.plos.org/plosone/s/file?id=wjVg/PLOSOne_formatting_sample_main_body.pdf and 
   <https://journals.plos.org/plosone/s/file?id=ba62/PLOSOne_formatting_sample_title_authors_affiliations.pdf\>

**We have aligned our file naming with the guidelines.**

2. Please provide additional details regarding participant consent. In the ethics statement in the Methods and online submission information, please ensure that you have specified what type you obtained (for instance, written or verbal, and if verbal, how it was documented and witnessed). If your study included minors, state whether you obtained consent from parents or guardians

**We have updated the relevant part of the manuscript (lines 225-231). Written consent was obtained from all pregnant women and midwives. The consent forms used were approved by the ethics committee.**

3. Context
   a. There are limited data on TB screening in pregnancy but the authors omit some previous studies and do not provide detail with respect to WHO recommendations:
      Schwartz, et al. Pasipamire et al. (reviewer 2), Ranaivomanana et al. (Reviewer 3) and Hoffman et al. (doi:10.1371/journal.pone.0062211)
      The latter found a very low sensitivity for the WHO 4-symptom screen in pregnancy which should be incorporated into your discussion/limitations. In addition, GeneXpert screening at first antenatal visit has recently been introduced in South Africa (the symptom screen has been standard of care for some time) and there may be additional data available from this country. GeneXpert may be less sensitive when used as a screening test as opposed to a diagnostic test. However, here it is used for confirmation. Prof Neil Martinson has done a lot of work in this regard.

**We have included further discussion of previous studies and WHO recommendations in our introduction (lines 62-78).**

b. The information about TB and HIV in Benin and Conotou is helpful to situate the study; antenatal HIV prevalence rates would be relevant. Most TB in sub-Saharan Africa presents in people living with HIV. HIV status should also be presented in the results and noted in the discussion.

**We have included mention of HIV status of those with symptoms and those who tested positive for TB in our results and speculated on the role of HIV in the discussion section (lines 273-279; 361-370).**

4. Interpretation: The antenatal TB prevalence was relatively low, although higher than that in the general population. Please note the concerns of reviewers 1,2 and 3 with respect to screening and timepoints. Some of these comments can be addressed as limitations. Most maternal TB presents in the post-partum period and screening in this population may be useful. There is also South African data on TB prevalence in pregnancy that may be useful. It is not clear that the infant death was related to maternal TB. Be careful of making this assumption without evidence.

**We have amended our wording and clarified the timepoints and interpretation of screening. We have also carefully worded our interpretation of the infant death, in response to these comments (lines 390-392).**

5. Qualitative Component Did you use a software package to assist with analysis? Reporting of the qualitative results is inadequate (reviewer 1).

**We did not use a software package to assist with analysis of qualitative data (lines 187). We have improved our reporting of qualitative data in response to the reviewer comments (lines 286-335).**

**Reviewer Scores**

1. Is the manuscript technically sound, and do the data support the conclusions?

The manuscript must describe a technically sound piece of scientific research with data that supports the conclusions. Experiments must have been conducted rigorously, with appropriate controls, replication, and sample sizes. The conclusions must be drawn appropriately based on the data presented.

Reviewer #1: Partly

Reviewer #2: Partly

Reviewer #3: Yes

2. Has the statistical analysis been performed appropriately and rigorously?

Reviewer #1: No

Reviewer #2: No

Reviewer #3: I Don't Know

3. Have the authors made all data underlying the findings in their manuscript fully available?

The [PLOS Data policy](http://www.plosone.org/static/policies.action#sharing) requires authors to make all data underlying the findings described in their manuscript fully available without restriction, with rare exception (please refer to the Data Availability Statement in the manuscript PDF file). The data should be provided as part of the manuscript or its supporting information, or deposited to a public repository. For example, in addition to summary statistics, the data points behind means, medians and variance measures should be available. If there are restrictions on publicly sharing data—e.g. participant privacy or use of data from a third party—those must be specified.

Reviewer #1: No

Reviewer #2: No

Reviewer #3: Yes

4. Is the manuscript presented in an intelligible fashion and written in standard English?

PLOS ONE does not copyedit accepted manuscripts, so the language in submitted articles must be clear, correct, and unambiguous. Any typographical or grammatical errors should be corrected at revision, so please note any specific errors here.

Reviewer #1: Yes

Reviewer #2: Yes

Reviewer #3: Yes

**In response to the scores above, we have improved our reporting of methods and amended our conclusions. We’ve further elaborated on our statistical methods and provided justification or amended, where necessary. The underlying data used in this manuscript is de-identified data; the excel file containing this data and a data dictionary are included.**

**Reviewer #1**

Prevalence, acceptability and cost of routine screening for pulmonary tuberculosis
among pregnant women in Cotonou, Benin

Thank you for asking me to review the manuscript ‘Prevalence, acceptability and cost of routine screening for pulmonary tuberculosis among pregnant women in Cotonou, Benin.’ In this mixed-methods, cross-sectional study with a cost assessment, the authors evaluate the yield, cost, feasibility, and acceptability of routine tuberculosis (TB) screening of pregnant women in Cotonou, Benin. The manuscript is well-written and on the whole reads easily. However, I have some comments below which do need to be addressed before this manuscript can be published.

Main Comments

1. Prevalence rate
   a. The authors’ report a TB prevalence rate of 49 per 100,000 among pregnant women enrolled in their study. However, in this cross-sectional study ~ 4000 pregnant women were screened only once during their pregnancy for TB, and not throughout their pregnancy. In addition, only those who had a cough longer than two weeks were asked to provide sputum for analysis. So, in fact, are the authors not are reporting a prevalence rate of 49 per 100,000 for one visit amongst those who had a cough and for whom a sputum sample was sent? For a TB prevalence rate among pregnant women, all ~ 4000 pregnant women would need to be screened and tested at each and every ANC visit throughout their pregnancy. The authors do acknowledge in the limitations that participants were only screened once during their pregnancy, but the authors need to clarify that they are reporting a prevalence rate of 49 per 100,000 for one visit amongst those who had a cough and for whom a sputum sample was sent.

**Thank you for this suggestion to improve our reporting, we have added words to the methods and results to make clear what the prevalence rate represents.**

b. Given the limitations in using only two cases of TB to determine a prevalence rate, a more robust discussion of the sensitivity and specificity of a cough of longer than two weeks and GeneXpert would be helpful. Given the increasing awareness of asymptomatic TB, this must be addressed in the discussion.

**We agree that more could be said about both our symptom screening method and our diagnostic method and have added to the introduction section (lines 63-78) and discussion section (lines 361-370).**

2. The death of the infant in one of the women diagnosed with TB.
   In the all-important first paragraph of the discussion the authors write ‘the benefit of early detection and prompt treatment is apparent, given the adverse fetal outcome among one woman diagnosed with TB.’ The implication is that the infant’s death was TB-related. However, no evidence is given confirming this. Was the infant diagnosed with congenital TB? Were congenital or other morbidities excluded?

**We have clarified our wording here to remove ambiguity.**

3. Reporting of cost analysis
   Once published, this article will most probably be read by people who work in TB, many of whom may not be familiar with the technical terms used to describe the cost analysis. Simpler more accessible terminology should be used for the following:
   ‘purchasing power parity’
   ‘material costs’
   ‘accessioning’
   ‘annuitized’ – the term annualised is more intuitive.
   ‘prorated’
   ‘realized costs’

**Thank you for this suggestion. With respect to some terminology (PPP, material costs) we have kept this language as these are the only terms used—PPP is qualified as a currency conversion factor and material costs we believe are self-explanatory. We have clarified terminology with respect to accessioning (sample registration and preparation), replaced the term annuitized, clarified prorated (averaged across all patients), and clarified realized costs (those actually incurred).**

4. The reporting of the qualitative results (Page 10)
   ‘All pregnant women interviewed supported TB screening during routine antenatal care visits and
   mentioned its usefulness in preventing pregnancy complications. All midwives also found routine
   TB screening during antenatal care visits to be very useful…’
   This very generalised reporting of the qualitative results is inadequate and more detail is needed to substantiate the findings reported.
   • What do you mean pregnant women supported TB screening? What was said in the interviews that led you to this conclusion?
   • What pregnancy complications did pregnant women say could be avoided by screening?
   • What did the midwives say that led the authors to conclude that screening during ANC visits was useful?

**Thank you for the opportunity to elaborate. We have now included some example quotes (translated) to corroborate our conclusions. This is contained on lines 286-335.**

Minor Comments

5. Page 3:
   The sentence ‘Additional factors such as human immunodeficiency virus (HIV) and diagnosis of advanced disease at late term in pregnancy are also associated with poor prognosis in pregnant women with TB.’
   This sentence is unclear, are you referring to advanced TB or advanced other diseases?

**In our editing, we have elected to remove this sentence—initially we intended to mean advanced TB disease.**

6. Page 4:
   ‘…..located within a radius of 15km from the NTP and included three public…..’
   Do you mean the NTP offices?

‘Consecutive women…’ Do you mean all women?
‘Included women were between the ages of 14 and 45 years and had a pregnancy test, an ltrasound, or a gynecological examination certifying pregnancy…..’
Were these your inclusion and exclusion criteria? If so clarify this.

**The national tuberculosis programme is located inside a TB hospital, we have clarified this. When we say consecutive, we mean all women meeting inclusion criteria were screened for inclusion. We have clarified that these were indeed our inclusion criteria.**

7. Page 5:
   ‘Taking into consideration midwife workload, we asked them to at least focus on the most important sign which is the presence of a cough for more than 2 weeks.’
   After reading this sentence I was surprised to read in the results section that you did ask and record other symptoms. Maybe you could clarify in the text exactly what was done, something like: 'Having recorded all TB symptoms, the questionnaire focussed on coughs of more than 2 weeks.’

**We have edited this text. All women were asked about all 4 TB symptoms, but only those with cough of more than 2 weeks were then subjected to further questions and evaluation—this reduced the midwife workload and more closely reflected NTP guidance.**

8. Page 6:
   ‘All participants were randomly selected.’
   How were participants randomly selected?

‘The number of participants was determined a priori.’
On what basis was this a priori decision made.

‘These interviews were conducted onsite but separate from antenatal care rooms.’
What is of importance and interest is whether the interviews were conducted in a confidential place where the participant could not be overheard.

**We have clarified how we randomly selected the participants and clarified that the interviews were in a confidential place. We decided the number of participants based on what we deemed feasible.**

9. Page 9
   ‘Weight loss/stagnation’
   By stagnation do you mean a failure to gain weight?

**We have removed the term stagnation and replaced it with its definition.**

10. Page 12
    ‘….33% were premature…’
    This is unclear, you need to clarify that this was 33% of the infants.

**We have included the term ‘of infants’ in this sentence.**

**Reviewer #2**

Introduction:

1. Introduction does not mention previous studies that looked at TB screening in pregnancy e.g. Schwartz American Journal of perinatology 2009, Kosgei Public Health Action 2013, Feroz Ali International Journal of Infectious Diseases, Gounder JAIDS 2012, Pasipamire African Journal of Lab Medicine 2020 (These all include HIV-neg participants as well). I think its very important to look at what others have tried before even if it is in different settings - these differences can then be highlighted. You mention the WHO but dont telll us exactly what they recommend.

**We have added further context into what has been done in the past (lines 63-78).**

2. You should also provide some information about TB in Benin - Whats the prevalence in the general population and in pregnancy? If its not known then that should be said. What are the current procedures?

**We had included this information in the methods, where study setting is normally discussed. We have also included current practices for diagnosing TB in Benin in our methods (lines 90-104).**

3. This info could lead to why you are doing this study now? At the moment its hard to understand why this study is being done now because its not clear what we know and where the gaps in knowledge are.

**We have made clear the gap in knowledge being filled (lines 80-87).**

Methods:

4. Under study population, you mention ultrasound, gynae exam etc. Were these study procedures or part of routine care? I'm not sure if I missed it but are they relevant to the study - dont think you mention any of these results.

**These were inclusion/exclusion criteria for the study population. We have clarified.**

5. I think the decision to ask only about cough of 2 weeks is really important. The could affect the sensitivity and specificity. As it is, I think the 4 symptom screen might miss alot of cases especially in pregnancy when TB symptoms can be masked/atypical. The sens and spec etc of the screen should be mentioned and the possible effects on results discussed in the discussion. This is especially true when screening HIV positive pregnant women - do you know what proportion of the screened women had HIV?

**We agree that only further evaluating those with cough could reduce sensitivity, we had acknowledged this in our discussion. All 4 classic TB symptoms were asked for, but we only flagged those with cough for further evaluation as that is policy at the national level and it was deemed more feasible to implement. We only collected HIV status on those who had cough of at least 2 weeks.**

6. I was surprised to read under quantitative analysis that you would be comparing rates in pregnant and non-pregnant women as this had not been mentioned before. In the Introduction you mention Zenner et al. who showed higher TB rates in pregnant compared to non-pregnant populations, there are other studies also showing this - please explain to us in the intro why this needs to be done.

**We have clarified this. We compared it to the detected rate nationally in Benin for a comparable population group--detailed in our Methods. The introduction had stated that pregnant women were at increased risk for TB. If we could show similar in Benin, this would provide evidence that enhanced case finding at routine pregnancy visits could be more cost-effective than alternatives. We have made edits for clarity.**

7. I dont understand why you adjusted the estimate in non-pregnant females to 9 months since this was cross-sectional?

**The incidence of TB is stated over an annual time span (12 months). It represents a cumulative incidence of TB. A full term pregnancy lasts 9 months and thus, pregnant women are only eligible to contribute to the annual incidence for this time frame. To make the *follow-up times* in both groups equivalent (time frame they are eligible to be included as an incident case of TB) we adjusted the follow-up time for the comparator population to 9 months.**

8. Under qualitative analysis you say that sample size was decided a priori - what was this based on?

**We initially decided to include two women and two midwives per site in the qualitative interview. This was based on convenience and what the study team deemed feasible. This is stated explicitly now in our methods (lines 166-171)**

Results:

9. What was the HIV status of those with TB?

**We have clarified that both women were not living with HIV.**

Discussion:

10. In the first paragraph you say the benefits of early detection are clear because of the infant death but that does not make sense to me since this mother was screened but still had a poor outcome?

**Early detection *and treatment initiation* are important. Benefits of screening are lost if appropriate treatment is not given quickly. Treatment initiation was delayed for the women with an adverse fetal outcome. We recognize we may have overstated the benefits of screening based on the small sample size in the study and have removed this speculation with respect to our study, but keep the findings of Sobhy et al in our discussion.**

11. I dont understand what you are trying to say with the second paragraph? You say that TB symptoms are hidden in pregnancy (should reference this) but then go on to discuss the rate of cough, which may or may not be due to TB? I also thought that HIV positive women may be even less symptomatic than HIV-negative?

**We have edited this paragraph to be clear on our intended meaning and provide further context. We found a much lower prevalence of cough in our study than in others, and were speculating on possible mechanisms. Symptom screen sensitivity is lower among pregnant women, which is our intended meaning on symptoms being hidden.**

12. Last sentence of paragraph 3 - how was this calculated? It should be in the methods and results if you are reporting it.

**The data on the number of pregnancies was found from the Ministry of Health (now included in the reference). For such a rough calculation, we did not include this in the methods or results, as it was to give a feel on the overall budget impact of such a program. A formal budget impact analysis would need to be performed, in our opinion, to warrant inclusion in methods and results. The calculation was done by extrapolating the cost per pregnant woman screened and yield in our study to all pregnant women in Benin.**

13. Limitations - How would asking about only cough and not other symptoms affect results? I also wonder about the burden of asking about the additional 3 TB symptoms if you are already asking about the cough? How might that affected the cos-effectiveness ratio?

**We have clarified. We asked about all 4 TB symptoms, but only those with cough were flagged for further follow-up. We are unsure the added yield of further including those with any of the 4 TB symptoms—previous studies we referenced in our introduction suggest added symptoms have marginal benefit. We speculate that if we did Xpert testing on all women with any of the 4 symptoms, the yield would go up while the cost-per-TB case detected would increase by virtue of more people receiving Xpert (lines 425-427).**

14. You say knowledge of TB in pregnancy was low but this was not reflected in your results. In the results all you say is that knowledge of haemoptysis was low which is not really a common symptom. What was the basis for saying this?

**We have added further context to the qualitive analysis findings. In Benin, when patients present with hemoptysis, the initial reaction is to think TB. This is particularly important among persons with more advanced disease. We have elected to keep this statement.**

15. You say the cost per case detected was high compared to other interventions in Benin. What interventions were these?

**We have amended this sentence for clarity.**

**Reviewer #3**

1. The authors considered data from the Benin NTP with the number of notified cases of TB in 2016 and 2018 where 683 patients were diagnosed (results section) and from which the annual incidence for the study was deduced. In the calculations performed by the authors, were a distinction between pulmonary TB and EPTB data considered in the calculations? If so, mention please it in the materials and methods section, then discuss EPTBs effect in the prevalence observed in the discussions section.

**The number included counts all forms of TB – not just pulmonary. We have mentioned this now in the discussion section (lines 372-375). We speculate that prevalence may be even higher in pregnant women than we estimated as extrapulmonary TB would not generally be captured by our symptom screening algorithm.**

2. Was the obtained TB risk calculated obtained from all the participating pregnant women or is this risk observed only in those with among symptomatic pregnant women (cough lasting two weeks)? This has to be clarified in the manuscript.

**This came from all women in the study (0.05%)—the risk among those symptomatic was 2.3%.**

3. Limiting the inclusion to a two-week cough alone may be the cause of the lower than estimated outcome (as already cited in the discussions section). Recent studies in another country in sub-Saharan Africa, in an area where the HIV prevalence is lower than that of Benin, but with a higher incidence of TB showed a prevalence of 12.3% of TB in symptomatic pregnant women including coughs lasting more than two weeks but associated with other symptoms such as fever, chest pain, etc. (Ranaivomanana et al., 2021, IJTLD). Please confirm that the other symptoms of TB (fever, chest pain , loss of appetite, etc.) are not considered in the policies of the NTP in Benin as stated in the manuscript?

**The lead author (Dr. Adjobimey) is a representative of the NTP. We have clarified that the NTP prioritizes TB testing for those with cough over those with other TB symptoms.**

4. The low prevalence of cough found in the pregnant women of the study seems surprising knowing that the whole immune system of pregnant women is attenuated to protect the foetus. What steps did the authors take to ensure that the cough lasted at least two weeks? Is it by just verbally questionning the participants? How to confirm a two week cough? Please specify it in the manuscript.

**The duration of cough was self-reported after being questioned by the healthcare worker. We have clarified this.**

5. Since the prices displayed for laboratory tests are those of the GDF, it would be interesting to also give the % of each cost in the table (as in the text). This can be of interest to those who do not have access to the GDF program.

**We appreciate this thought, but are unsure of how to operationalize it without being misleading or making the table too busy. We feel that if readers are interested in the proportion of GeneXpert cost that is associated with the cartridge and/or equipment, we have provided all the information required for them to do such a calculation.**

6. Wouldn’ it be interesting to perform a DALY analyzis for this kind of study given the target population? It would be interesting to discuss about this.

**We have added a sentence on cost-utility analysis to our discussion (lines 411-413).**
